# Supplementary material for: Aleatoric uncertainty estimation with test-time augmentation for medical image segmentation with convolutional neural networks
Source: Neurocomputing (Amst). Author manuscript; Available in PMC 2019 Oct 8. (PMC6783308; doi:10.1016/j.neucom.2019.01.103)
Supplement: Supplementary Material [file EMS84271-supplement-Supplementary_Material.pdf]

Supplementary Material of "Aleatoric uncertainty estimation with test-time augmentation for medical image segmentation with convolutional neural networks"

Guotai Wang[a,b,c*], Wenqi Li[a,b], Michael Aertsen[d], Jan Deprest[a,d,e,f], Sébastien Ourselin[b], Tom Vercauteren[a,b,f]

[a] *Wellcome / EPSRC Centre for Interventional and Surgical Sciences, University College London, London, UK*
[b] *School of Biomedical Engineering and Imaging Sciences, King's College London, London, UK*
[c] *School of Mechanical and Electrical Engineering, University of Electronic Science and Technology of China, Chengdu, China*
[d] *Department of Radiology, University Hospitals Leuven, Leuven, Belgium*
[e] *Institute for Women's Health, University College London, London, UK*
[f] *Department of Obstetrics and Gynaecology, University Hospitals Leuven, Leuven, Belgium*

1. 2D Skin Lesion Segmentation

We further validated our proposed method with the International Skin Imaging Collaboration (ISIC) 2018 skin lesion segmentation dataset (Tschandl et al., 2018; Codella et al., 2018). Skin cancer is the most prevalent cancer in the United States where melanoma is the most dangerous type. Dermoscopy is a promising imaging technique for diagnosis of skin cancer (Siegel et al., 2017). Automatic assessment of dermoscopic images is attracting increasing attentions due to the shortage of dermatologists per capita. Segmentation of the lesion regions plays an important role for automatic measurement and diagnosis of skin cancer (Yuan et al., 2017).

*Corresponding author
Email address: `guotai.1.wang@kcl.ac.uk` (Guotai Wang[a,b,c])

1.1. Data and Implementation

We used the publicly available dataset of ISIC 2018 skin lesion segmentation challenge [1] (Tschandl et al., 2018; Codella et al., 2018). The lesion images were collected with a variety of dermatoscope types from several different institutions. Each image contained exactly one primary lesion, and smaller secondary lesions, other pigmented regions or other fiducial markers may be neglected. The released training dataset consisted of 2594 images with corresponding ground truth masks annotated by human experts. We randomly split them into 2000 images for training, 294 images for validation and 300 images for testing. We resized these images into a consistent size 192×192.

For experiments, we used 2D U-Net (Ronneberger et al., 2015) and Dense U-Net (Guan et al., 2018) that is an extension of U-Net with dense connection blocks. The networks were implemented in TensorFlow[2] (Abadi et al., 2016) using NiftyNet[3] (Li et al., 2017; Gibson et al., 2018). During training, we used Adaptive Moment Estimation (Adam) to adjust the learning rate that was initialized as 10^{-3}, with batch size 10, weight decay 10^{-7} and iteration number $20k$. We represented the transformation parameter $\boldsymbol{\beta}$ in the proposed augmentation framework as a combination of f_l, r and s, where f_l is a random variable for flipping in 2D, r is the rotation angle in 2D, and s is a scaling factor. The prior distributions of these parameters were modeled as: $f_l \sim Bern(0.5)$, $r \sim U(0, 2\pi)$, $s \sim U(0.8, 1.2)$ and $e \sim N(0, 0.05)$. We used data augmentation at both training and test time based on this formulation.

1.2. Segmentation Results with Uncertainty

Fig. 1 shows a visual comparison of different types of uncertainties for segmentation of skin lesion. The results were based on the same trained model of Dense U-Net, and the Monte Carlo simulation number N was 40 for TTD, TTA, and TTA + TTD to obtain *epistemic*, *aleatoric* and hybrid uncertainties respectively. The subfigures show three cases with different skin lesion sizes and appearances. In Fig. 1 (a), the first row presents the input and the segmentation obtained by the single-prediction baseline. The other rows show the three types of uncertainties and their corresponding segmentation results respectively. It can be observed that the TTD-based *epistemic*

[1] `https://challenge2018.isic-archive.com`
[2] `https://www.tensorflow.org`
[3] `http://www.niftynet.io`

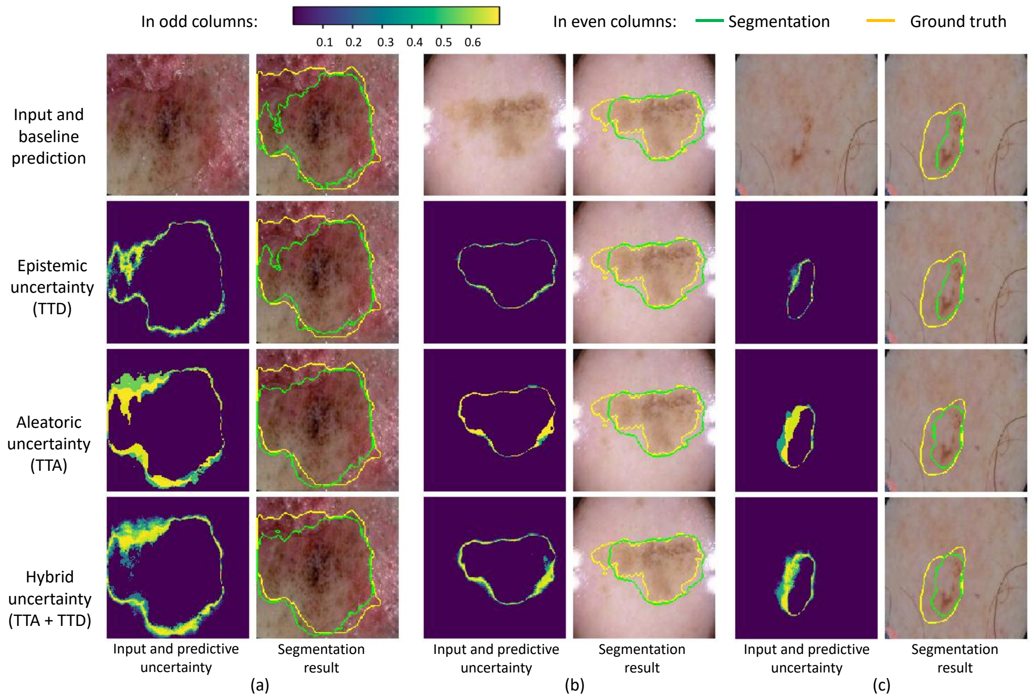

Figure 1: Visual comparison of different types of uncertainties and their corresponding segmentations of skin lesion. In the second column of each subfigure, the first image shows segmentation by the single-prediction baseline, and the others are based on Monte Carlo simulation with $N = 40$. TTD: test-time dropout, TTA: test-time augmentation.

uncertainty map mainly highlights the border of the segmented foreground. In contrast, the TTA-based *aleatoric* uncertainty map shows uncertain segmentations not only on the border but also in some challenging areas in the left up corner of the image. It can be observed that both the TTA-based *aleatoric* and hybrid uncertainty maps have a better performance in indicating potential mis-segmentations than the TTD-based *epistemic* uncertainty.

1.3. Quantitative Evaluation

To quantitatively evaluate the segmentation results, we measured Dice score and ASSD of each prediction for different testing methods of baseline single prediction, TTD, TTA and TTA + TTD. We also compared training with and without data augmentation. We found the Monte Carlo sample number N that obtained the performance plateau was 40. Table 1 shows the quantitative evaluation results for these different testing methods when N

Table 1: Dice (%) and ASSD (pixels) evaluation of 2D skin lesion segmentation by different training and testing methods. Tr-Aug: Training without data augmentation. Tr+Aug: Training with data augmentation.* denotes significant improvement from the baseline of single prediction in Tr-Aug and Tr+Aug respectively (p-value < 0.05). † denotes significant improvement from Tr-Aug with TTA + TTD (p-value < 0.05).

Train	Test	Dice (%)		ASSD (pixels)	
		U-Net	Dense U-Net	U-Net	Dense U-Net
Tr-Aug	Baseline	84.67±16.55	85.83±13.99	6.20±6.71	5.63±5.49
	TTD	84.91±16.23	86.02±13.94	6.13±6.62	5.62±5.45
	TTA	85.32±16.19*	86.48±13.67*	5.74±6.19*	4.82±5.36*
	TTA + TTD	**85.63±15.89***	**86.77±13.32***	**5.71±6.26***	**4.79±5.23***
Tr+Aug	Baseline	85.73±15.02	86.30±13.72	5.75±5.67	5.45±5.93
	TTD	85.95±14.94	86.48±13.81	5.72±5.61	5.36±5.66
	TTA	86.42±14.82*	87.02±13.65*	5.19±5.30*	4.39±4.87*
	TTA + TTD	**86.56±14.55***†	**87.11±13.47***	**5.15±5.26***†	**4.37±4.84***

was 40. For both networks we found that TTA led to a higher improvement of segmentation accuracy than TTD.

1.4. Correlation between Uncertainty and Segmentation Error

We also investigated the correlation between prediction uncertainty and segmentation error. For pixel-level evaluation, we measured the joint histogram of pixel-wise uncertainty and pixel-wise error rate for TTD, TTA, and TTA + TTD respectively, and the joint histograms were normalized by the overall pixel number in test images. Fig. 2 shows the results based on Dense U-Net using training with data augmentation and N set as 40. For each type of uncertainties, we calculated the average error rate at each uncertainty level, and obtained a curve of error rate as a function of uncertainty, i.e., the red curves in Fig. 2. This figure shows that when the uncertainty increases, the error rate also becomes higher gradually. The curves in Fig. 2(b) and Fig. 2(c) have higher slopes than that in Fig. 2(a), showing that TTA has fewer overconfident incorrect predictions than TTD and a better correlation with mis-segmentations.

For structure-level evaluation, we measured structure-level uncertainty represented by volume variance coefficient (VVC) and structure-level error represented by 1 - Dice. Fig. 3 shows their joint distributions with three different testing methods using 2D Dense U-Net that was trained with data augmentation. The Monte Carlo sample number was 40. The figure shows

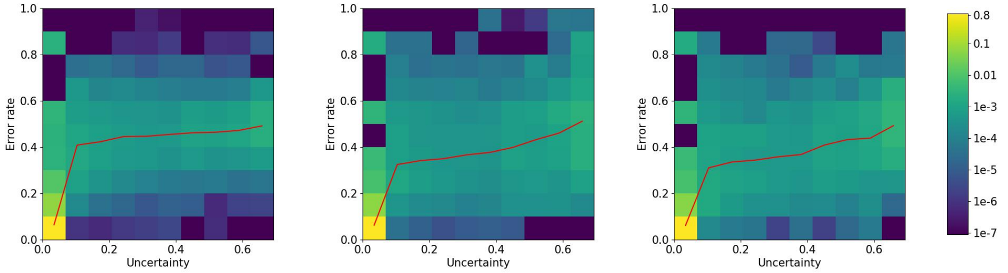

(a) TTD-based uncertainty (epistemic)

(b) TTA-based uncertainty (aleatoric)

(c) TTD + TTA-based uncertainty (hybrid)

Figure 2: Normalized joint histogram of prediction uncertainty and error rate for 2D skin lesion segmentation. The average error rates at different uncertainty levels are depicted by the red curves.

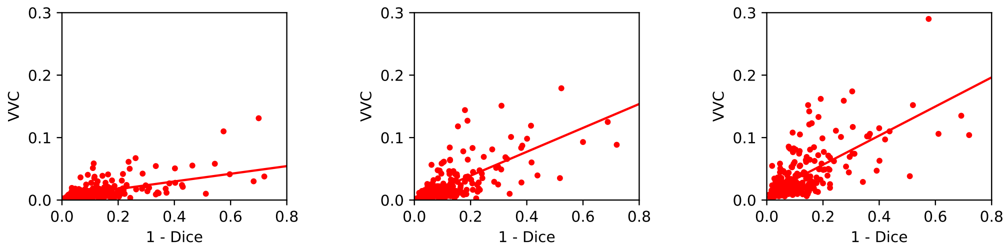

(a) TTD

(b) TTA

(c) TTA + TTD

Figure 3: Structure-level uncertainty in terms of volume variation coefficient (VVC) vs 1 - Dice for different testing methods in 2D skin lesion segmentation.

that for all the three types of testing methods, the achieved structure-level uncertainties increase with 1 - Dice. However, TTA-based testing has a larger slope than TTD-based testing, as shown in Fig. 3(a) and (b). TTA + TTD obtained similar results compared with TTA, as shown in Fig. 3(c).

References

Abadi, M., Barham, P., Chen, J., Chen, Z., Davis, A., Dean, J., Devin, M., Ghemawat, S., Irving, G., Isard, M., Kudlur, M., Levenberg, J., Monga, R., Moore, S., Murray, D.G., Steiner, B., Tucker, P., Vasudevan, V., Warden, P., Wicke, M., Yu, Y., Zheng, X., Brain, G., 2016. TensorFlow: A system for large-scale machine learning, in: USENIX Symposium on Operating Systems Design and Implementation, pp. 265–284.

Codella, N.C.F., Gutman, D., Celebi, M.E., Helba, B., Marchetti, M.A., Dusza, S.W., Kalloo, A., Liopyris, K., Mishra, N., Kittler, H., Halpern, A., 2018. Skin Lesion Analysis Toward Melanoma Detection: A Challenge at the 2017 International Symposium on Biomedical Imaging (ISBI), Hosted by the International Skin Imaging Collaboration (ISIC), in: IEEE International Symposium on Biomedical Imaging, pp. 168–172. 1710.05006.

Gibson, E., Li, W., Sudre, C., Fidon, L., Shakir, D.I., Wang, G., Eaton-Rosen, Z., Gray, R., Doel, T., Hu, Y., Whyntie, T., Nachev, P., Modat, M., Barratt, D.C., Ourselin, S., Cardoso, M.J., Vercauteren, T., 2018. NiftyNet: A deep-learning platform for medical imaging. Computer Methods and Programs in Biomedicine 158, 113–122.

Guan, S., Khan, A., Chitnis, P.V., Sikdar, S., 2018. Fully Dense UNet for 2D Sparse Photoacoustic Tomography Reconstruction. arXiv 1808.10848.

Li, W., Wang, G., Fidon, L., Ourselin, S., Cardoso, M.J., Vercauteren, T., 2017. On the compactness, efficiency, and representation of 3D convolutional networks: brain parcellation as a pretext task, in: International Conference on Information Processing in Medical Imaging, pp. 348–360.

Ronneberger, O., Fischer, P., Brox, T., 2015. U-Net: Convolutional networks for biomedical image segmentation, in: International Conference on Medical Image Computing and Computer-Assisted Intervention, pp. 234–241.

Siegel, R.L., Miller, K.D., Jemal, A., 2017. Cancer Statistics, 2017. CA: a cancer journal for clinicians 67, 7–30. arXiv:1011.1669v3.

Tschandl, P., Rosendahl, C., Kittler, H., 2018. Data descriptor: The HAM10000 dataset, a large collection of multi-source dermatoscopic images of common pigmented skin lesions. Scientific Data 1803.10417.

Yuan, Y., Chao, M., Lo, Y.C., 2017. Automatic Skin Lesion Segmentation Using Deep Fully Convolutional Networks with Jaccard Distance. IEEE Transactions on Medical Imaging 36, 1876–1886. 1807.06466.
